# Clinical applications of fibroblast activation protein inhibitor positron emission tomography (FAPI-PET)

**DOI:** 10.1038/s44303-024-00053-z

**Published:** 2024-11-13

**Authors:** Yuriko Mori, Emil Novruzov, Dominik Schmitt, Jens Cardinale, Tadashi Watabe, Peter L. Choyke, Abass Alavi, Uwe Haberkorn, Frederik L. Giesel

**Affiliations:** 1 Department of Nuclear Medicine, Medical Faculty and University Hospital Duesseldorf, Heinrich-Heine-University Duesseldorf, Duesseldorf, Germany; 2 Institute for Radiation Sciences, Osaka University, Osaka, Japan; 3 Molecular Imaging Branch, Center for Cancer Research, National Cancer Institute, National Institutes of Health, Bethesda, MD USA; 4 Department of Radiology, Hospital of University of Pennsylvania Philadelphia, Philadelphia, PA USA; 5 Department of Nuclear Medicine, Heidelberg University Hospital, INF 400, Heidelberg, Germany

**Keywords:** Cancer, Oncology

## Abstract

The discovery of fibroblast activation protein inhibitor positron emission tomography (FAPI-PET) has paved the way for a new class of PET tracers that target the tumor microenvironment (TME) rather than the tumor itself. Although ^18^F-fluorodeoxyglucose (FDG) is the most common PET tracer used in clinical imaging of cancer, multiple studies have now shown that the family of FAP ligands commonly outperform FDG in detecting cancers, especially those known to have lower uptake on FDG-PET. Moreover, FAPI-PET will have applications in benign fibrotic or inflammatory conditions. Thus, even while new FAPI-PET tracers are in development and applications are yet to enter clinical guidelines, a significant body of literature has emerged on FAPI-PET, suggesting it will have important clinical roles. This article summarizes the current state of clinical FAPI-PET imaging as well as potential uses as a theranostic agent.

## Introduction

The importance of the tumor microenvironment (TME) and its influence on tumor immunity has been a dominant theme of cancer research for the last decade. Multiple immune-modulating therapies have been introduced including checkpoint inhibitors, chimeric antigen receptor T cells (CAR-T), cancer vaccines and T cell transfer therapy, among others, which depend on activating the anti-tumor immune response. These therapies can lead to dramatic clinical responses^1^. Cancer-associated fibroblasts (CAFs) are stromal cells found in the TME of some tumors. They represent the “activated state” of tissue fibroblasts and are known to aid cancer growth^2^ by interfering with immune surveillance while producing stromal proteins that physically isolate cancer cells from exposure to the immune response and drug therapy. Indeed, the full range of pro-tumor effects of activated fibroblasts is still not completely understood. CAFs exhibit a specific membrane expression profile including the expression of fibroblast activation protein (FAP). Recent advances in cell biology and radiochemistry have enabled the development of radiolabeled FAP inhibitors that bind to membrane-bound FAP with high affinity. The original compounds have now grown into a class of FAP-targeting ligands and permit the non-invasive imaging of FAP expression in vivo.

In this review we provide an overview of the current status of FAPI-PET, beginning with a brief description of FAP and FAP tracers, the importance of tumor stroma, and the potential clinical applications of this new class of PET tracers in comparison to FDG-PET. Here, we summarize the current understanding of FAPI-PET in selected diseases.

## Stromal targeting with FAP

### Fibroblast activation protein (FAP)

Fibroblast activation protein (FAP) was initially described by Wolfgang Rettig in 1988 as a cell surface antigen expressed on reactive stromal fibroblasts within many epithelial cancers and in granulation tissue of wound healing, as well as on tumor cells of many sarcomas^3^. Conversely, FAP expression is absent in most normal tissue, including normal fibroblasts, non-malignant epithelial cells, or the stroma of benign epithelial tumors^3^.

FAP was subsequently identified as a membrane-bound type II serine protease^4,5^, which is chemically classified as a member of the dipeptidyl peptidase IV (DPPIV) family of proteins. FAP consists of 760 amino acids with a short intra- (6 amino acids), trans- (20 amino acids), and a large extracellular membrane domain (734 amino acids)^6^, the latter offering a favorable docking site for ligands^7,8^. FAP possesses both endo- and exopeptidase activities and, thus, enables matrix remodeling through the cleavage of matrix proteins^9,10^. FAP is overexpressed in CAFs, which can be found in over 90% of epithelial cancers to varying degrees^10^. For instance, CAFs are commonly found in abundance in neoplasms with strong desmoplastic reactions such as pancreatic^11,12^, colorectal^13,14^, and breast^15–17^ cancer. The possible association of FAP expression with tumor aggressiveness was noted even in the early literature^13,18,19^ and subsequently confirmed in various cancer types^3,11–23^. Moreover, FAP can also be expressed directly in a limited number of cancer cells, such as some ovarian^20–22^, breast^15,16^, an pancreatic cancers^12,24^ as well as in sarcomas^3,23^, suggesting possible theranostic applications in the future.

### FAP-targeting tracer

Small enzyme inhibitors specific for FAP were initially proposed by Jansen et al, who designed several compounds including UAMC-1110, which showed specificity for FAP^25,26^. Through the chemical modification of the quinoline group of UAMC-1110, researchers at the University of Heidelberg successfully synthesized a family of FAPI tracers beginning in 2018^27^. These compounds demonstrated inhibition of the endopeptidase activity of FAP. Moreover, the attachment of 1,4,7,10-tetraazacylclododecane-1,4,7,10-tetrayl tetraacetic acid (DOTA)-chelator enabled binding of Ga-68 for PET imaging, as well as other therapeutic radioisotopes such as Lu-177^27^. In the initial production of FAPI tracers, FAPI-04 revealed the most favorable pharmacokinetic profile with higher uptake and more tumor retention up to 3 h post injection^28^. Dosimetric analysis showed a suitable equivalent dose of approximately 3–4 mSv for 200 MBq, which is comparable to ^18^F-FDG or ^68^Ga-DOTA-(Tyr3)-octreotate (DOTATATE)^28^. In the subsequent series of tracer development, ^68^Ga-FAPI-46 showed even higher tumor retention and tumor-to-background ratio (TBR)^29^. Currently, these tracers namely ^68^Ga-FAPI-04 and -46, are the most widely used FAP tracers around the world^30^. While ^68^Ga-labeling has inherent limitations due to its shorter half-life and limited synthesis capacity, ^18^F- or ^99m^Tc-labeled FAPI compounds (^18^F-FAPI-74 for PET, ^99m^Tc-FAPI-34 for SPECT imaging) have also been synthesized^31–33^. These compounds provide potential advantages for wider clinical use because of the longer half-life of these isotopes. For instance, labeling with F-18 enables a larger production batch which can then be distributed around a metropolitan area rather than requiring labeling at each imaging site. ^99m^Tc with its 6 h half-life utilizes local labeling but enables the application of SPECT cameras instead of PET, which could reduce costs^34^.

**Fig. 1:**
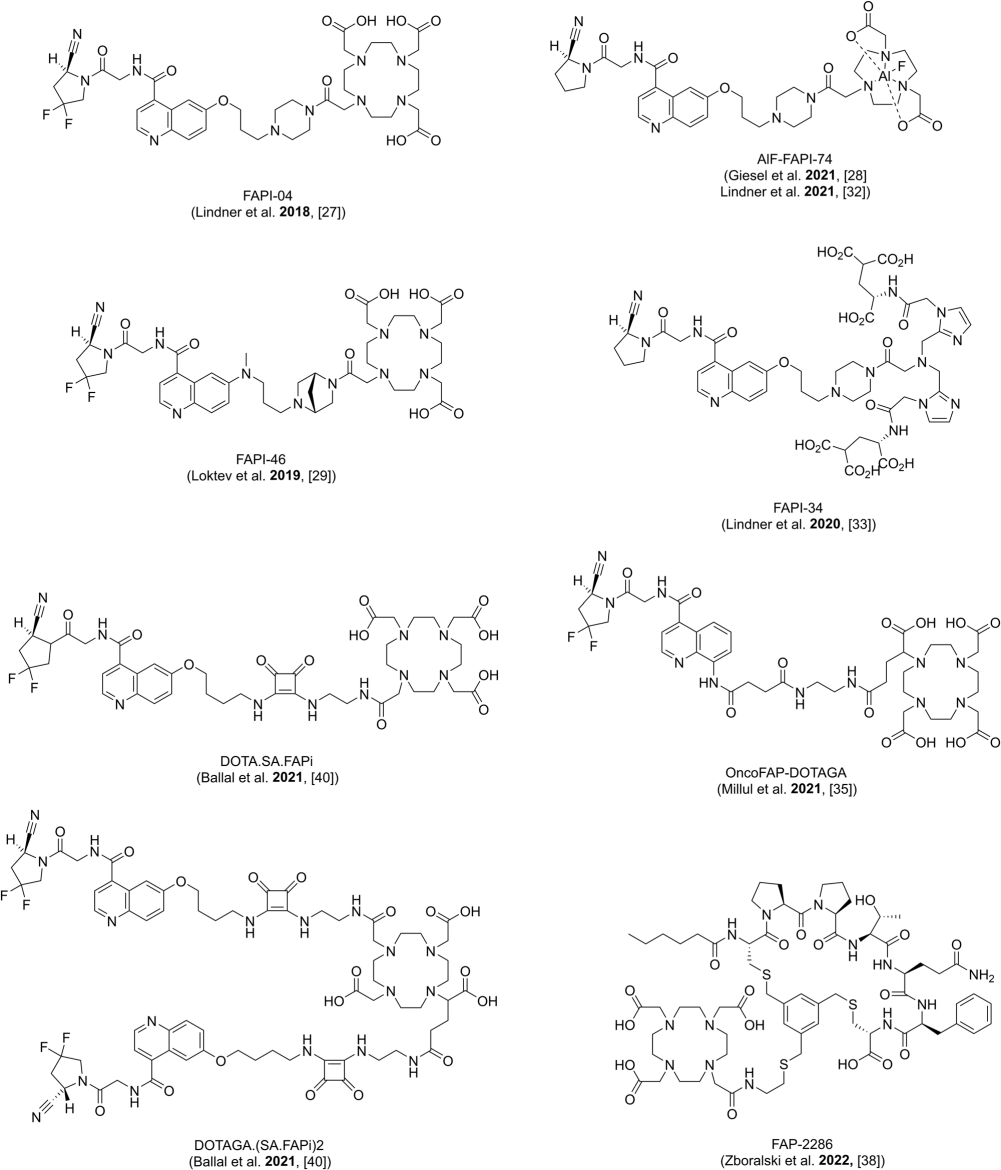
Chemical structures of the selected FAP tracers. Chemical structures of the selected FAP tracers for diagnostic use, except (DOTAGA.(SA.FAPi)_2_, which is the dimer of the diagnostic tracer DOTA.SA.FAPi and more suitable for therapeutic purposes due to the longer tumor retention.

Other promising approaches to targeting FAP include targeting peptides or peptidomimetic compounds^35,36^. OncoFAP is a small organic FAP ligand with ultrahigh affinity^35,37^. Baum and colleagues presented a new cyclic peptide FAP-2286 that selectively binds to FAP with low off-target activity^36,38^. Further modification of the quinoline-based structure has been performed by Ballal and colleagues, who developed ^68^Ga-DOTA.SA.FAPi as a quinoline-based monomer which offers the possibility of wider theranostic use through dimerization (DOTAGA.(SA.FAPi)_2_^39,40^. The chemical structures of the selected FAP tracers are shown in Fig. 1.

## Oncological indications

### Overview

^18^F-FDG is currently the most widely used radiotracer in oncological imaging^41^, however, it has well known limitations^42–44^. For instance, FDG accumulates only in glucose-consuming cells and many cancers are either metabolically inactive or rely on energy sources other than glucose^42^. Moreover, FDG is also taken up in inflammation, which can lead to uncertainty over whether residual uptake is due to persistent tumor or inflammatory response to therapy^45^. FDG is also subject to variability due to patient preparation which includes fasting and resting prior to scanning^42^. In contrast, FAPI-PET is independent of glucose metabolism and there is no need for patient preparation. Whereas an incubation period of 60 min is recommended for FDG-PET^45^, FAPI-PET can be initiated as early as 10 min post injection (p.i.) with an acceptable imaging quality up to 3 h p.i^28,46^. Whereas FDG can be retained in background tissues, biodistribution studies of ^68^Ga-FAPI-PET show fast renal clearance and low tracer uptake in normal organs^28^. This results in significantly lower background signal compared to FDG, especially in FDG-avid organs such as brain, liver, or gastrointestinal tract^28^. Lower background results in higher target-to-background ratios (TBRs) with improved image contrast, contributing to the higher sensitivity for detecting malignant lesions that has been observed in many comparisons studies^43,44,47,48^.

### Cancer-associated fibroblasts (CAFs) and stroma

**Fig. 2:**
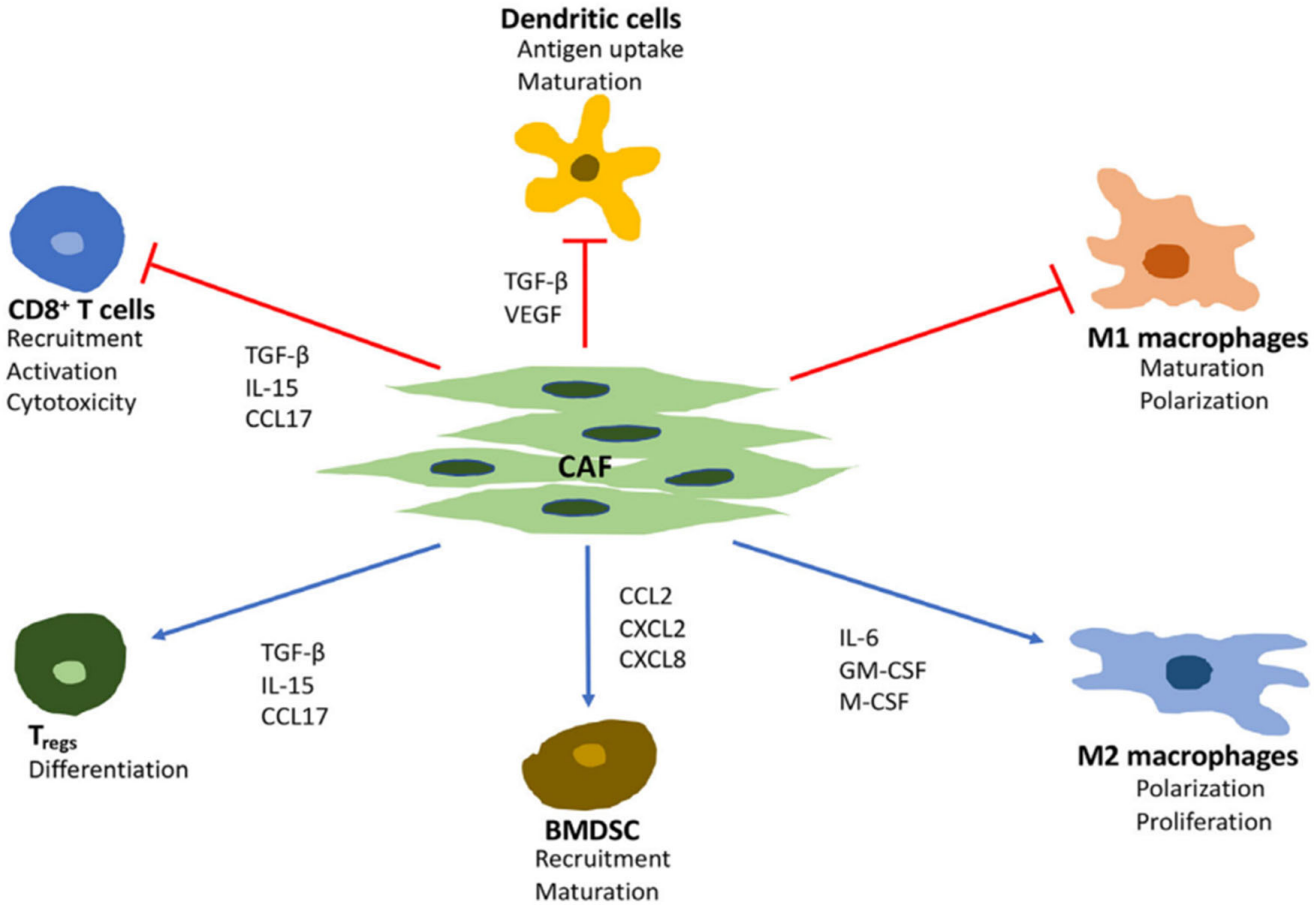
Effects of cancer-associated fibroblast (CAF) on immune cells. Major effects of cancer-associated fibroblast (CAF) on immune cells in the tumor microenvironment. TGF-β transforming growth factor beta, VEGF vascular endothelial growth factor, IL interleukin 6, GM-CSF granulocyte-macrophage colony-stimulating factor, M-CSF macrophage colony-stimulating factor, CCL C-C motif chemokine ligand, CXCL C-X-C motif ligand. BMDSC bone marrow-derived suppressor cells, Tregs regulatory T cells. [From ref. ^244^].

To be an effective diagnostic biomarker, FAPI-PET requires that tumors contain FAP-expressing CAFs in the tumor stroma or on the tumor cells themselves. Stroma typically consists of various immune cells, fibroblasts, endothelial cells, and extracellular matrix (ECM), surrounding neoplastic cells^19,49,50^ and is present even in small tumors of 1–2 mm in diameter^51^. Cancer-associated fibroblasts (CAFs) are often the most abundant cell type in the TME^19,50^ and arise mainly from normal, resting fibroblasts^19,52,53^. However, other precursors of CAFs include hematopoietic stem cells, as well as epithelial and endothelial cells^54–58^. CAFs interact with other key immunomodulatory cells including tumor‐associated macrophages (TAMs), regulatory T cells (Tregs), and myeloid‐derived suppressor cells (MDSCs) by releasing growth factors and proinflammatory cytokines, such as transforming growth factor β (TGF-β), vascular endothelial growth factor (VEGF), and interleukin-6 (IL-6)^50,59–65^. For example, in some breast cancers CAFs play an immunosuppressive role by promoting monocyte migration and transforming macrophages to the M2-subtype using monocyte chemotactic protein‐1 (MCP‐1) and stromal cell‐derived factor‐1 (SDF‐1)^59^. Hence, CAFs emerge as a potential target for immune modulation^59,66^. The interactions of FAP with various immune cells in TME are schematically illustrated in Fig. 2^64^.

When resident tissue fibroblasts convert to CAFs they morph from spindle- to stellate-shape and begin to express new surface markers, including α-smooth muscle actin (α-SMA), platelet-derived growth factor receptor (PDGFRα/β), vimentin, and FAP^15,67–69^, among which FAP is the most specific^49,67^ (Table 1). This activation can be caused by growth factors, such as TGF-β^70,71^. CAFs are actually quite heterogeneous with diverse functions, leading to several subclassifications^68,72,73^. Two commonly identified subpopulations of CAFs, ‘”myoCAFs” and “iCAFs”, are based on whether the CAF exhibits a matrix-producing contractile phenotype (myoCAF) or an immunomodulating phenotype (iCAF), respectively^67,74^. In pancreatic cancer, CAFs near the cancer cells exhibit a myoCAF phenotype with high TGF-β-driven α-SMA expression and lower levels of IL-6 (α‐SMAhighIL‐6low), while the more peripheral CAFs have lower αSMA expression with higher levels of IL-6 (α‐SMAlowIL‐6high) consistent with iCAFs^74^. The potential impact of these CAF subpopulations on tumor growth and on FAP imaging is still unknown and needs further elucidation.

**Table 1:** Markers of CAF

Activated CAFs		Quiescent CAFs
Commonly used markers	α-SMA	Vimentin
	FSP-1	
	FAP	
Rarely used markers	Tenascin-C	
	Periostin	
	NG-2	
	Desmin	
	PDGFR-α	
	PDGFR-β	
	Thy-1	
	Podoplanin	
	Paladin	
Negative markers	Cytokeratin	
	CD31	

[From ref. ^50^]

### Liver cancer

About half of hepatocellular carcinomas (HCC) are hypo- or isometabolic compared to normal liver and thus, show poor FDG uptake^75^. Diminished FDG uptake is due to high FDG-6 phosphatase activity and low glucose transporter expression, generally found in moderate to well-differentiated HCC^76^. Thus, FDG-PET is of limited value in HCC management.

Multiple studies comparing FDG-PET to FAPI-PET in HCC have documented the superior sensitivity and TBR of FAPI-PET^77–82^. For instance, the sensitivity of FAPI-PET for HCC varies in studies from 96 to 100% compared to 50–80% for FDG imaging^77–80^. The difference in sensitivity is especially pronounced for small lesions (≤2 cm in diameter) where the sensitivity of FAPI-PET vs. FDG-PET was 69% vs. 19% respectively and for well- or moderately differentiated vs. poorly differentiated HCCs where the difference was 83% vs. 33% respectively^81^. Higher SUVmax and TBR values for HCC have been found with ^18^F-FAPI-74 PET compared to ^18^F-FDG-PET (SUVmax: 6.7 vs. 4.3, TBR: 3.9 vs. 1.7, both *P* < 0.0001)^82^. This study also documented a higher detection rate for intrahepatic lesions (92.2% vs 41.1%, *P* < 0.0001) and lymph node metastases (97.9% vs 89.1%; *P* = 0.01), while the detection of distant metastases was more comparable (63.6% (42/66) vs 69.7% (46/66), *P* > 0.05)^82^. FAPI-PET resulted in upstaging, leading to changes in therapy planning in 48% of patients^82^. FAPI-PET sensitivity (96%) has also been favorably compared to other modalities such as MRI (100%) or contrast-enhanced CT for detection of HCC (96%) in comparison with FDG-PET (65%)^77^. The specificity of FAPI-PET varies from 90–100%.

Thus, FAPI-PET appears to be a highly sensitive and specific method of identifying HCC and is superior in sensitivity and specificity to FDG-PET. It will likely prove to be very helpful in identifying recurrences after focal therapy of HCC and thus, could become an important modality in the management of patients with HCC.

### Biliary tract cancer

Cholangiocarcinoma (CCC) is characterized by a strong desmoplastic reaction similar to that seen in pancreatic cancer^83^, making FAPI-PET a promising modality for detecting this cancer. Studies have documented that FAPI-PET has superior sensitivity to FDG-PET for primary CCC (98% vs. 86%), lymph node metastases (90% vs. 87%), and distant metastases (100% vs. 84%)^84^. Higher tracer uptake was observed for FAPI-PET compared to FDG-PET for intrahepatic lesions, pelvic nodal metastases, and distant metastases such as in the pleura or omentum^84,85^. FAPI-PET led to changes in tumor staging in many patients^85^.

A possible correlation between FAPI uptake and the grade of malignancy has been suggested by several authors^84–86^. For instance, Pabst et al. demonstrated that Grade 3 CCC tumors showed a significantly higher ^68^Ga-FAPI-46 uptake than grade 2 tumors (SUVmax 12.6 vs. 6.4; *P* = 0.009)^86^. FAP expression was high in the tumor stroma of CCC (∼90% of cells were positive)^86^. Furthermore, FAPI-PET uptake correlated with carcinoembryonic antigen (CEA) and carbohydrate antigen19-9 (CA19-9), which are prognostic biomarkers^84^.

Thus, FAPI-PET demonstrates superior sensitivity to FDG-PET for CCC including at the localized, locally advanced, and metastatic stages of the disease.

### Gastric cancer

**Fig. 3:**
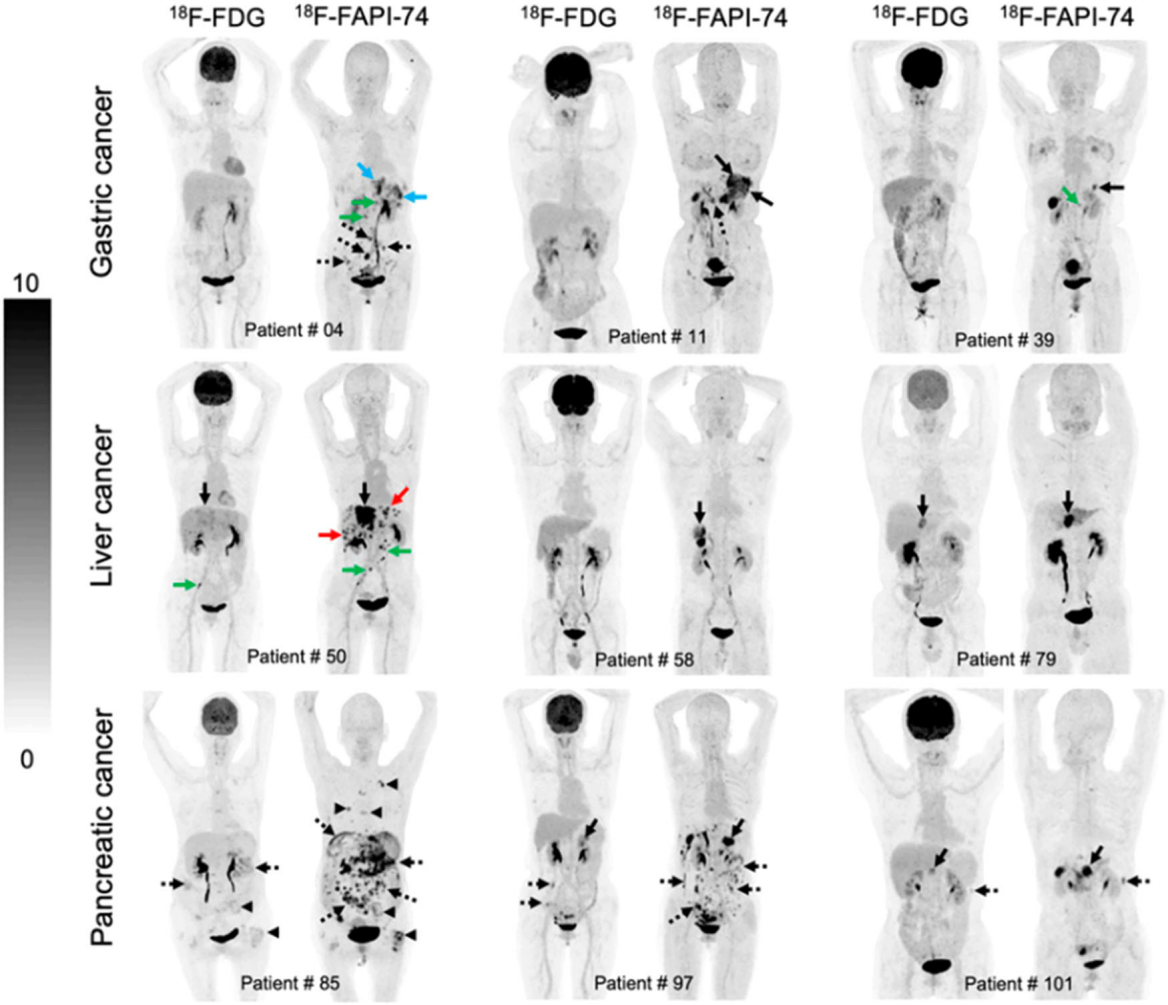
Head-to-head comparison of ^18^F-FDG and ^18^F-FAPI-74 PET imaging in selected oncologic patients. Nine representative oncologic patients who underwent ^18^F-FDG and ^18^F-FAPI-74 PET imaging. ^18^F-FAPI-74 PET outperforms ^18^F-FDG PET in detecting primary tumors (patients 11, 39, 50, 58, 79, and 101; solid black arrows), local recurrences (patient 4; blue arrows), abdomen lymph node metastases (patients 4 and 50; green arrows), intrahepatic metastases (patient 50; red arrows), bone metastases (patient 85; arrowheads), and peritoneal metastases (patients 4, 11, 85, 97, and 101; dotted arrows). [From ref. ^88^].

In general, FDG-PET has low uptake in many gastric cancers including signet-ring cell carcinoma, mucinous adenocarcinoma, and non-interstitial diffuse type cancer^87^. In such patients, FAPI-PET has shown superior sensitivity to FDG-PET^88–95^ (Fig. 3). For instance, in gastric signet-ring cell carcinoma (GSRCC), FAPI-PET showed a higher detection rate of primary lesions (73% vs. 18%), lymph nodes (77% vs. 23%), and distant metastasis (93% vs. 39%)^91^. SUVmax and TBR values of ^68^Ga-FAPI-PET were significantly higher than ^18^F-FDG-PET in primary tumors (SUVmax: 5.2 vs. 2.2; TBR: 7.6 vs. 1.3, *P* < 0.001), lymph nodes (SUVmax: 6.8 vs. 2.5; TBR: 5.8 vs. 1.3, both *P* < 0.001), and bone and visceral metastases (SUVmax: 6.5 vs. 2.4; TBR: 6.3 vs. 1.3, both *P* < 0.001)^91^. In a cohort with mixed gastric cancer subtypes, three studies confirmed superior sensitivity of FAPI-PET over FDG-PET (88% vs. 60%)^88–90^.

Despite these favorable results, other studies have reported limitations of FAPI-PET in assessing lymph node status in gastric cancer^92,95^, probably due to the heterogeneous nature of these tumors. For example, one study found no difference in sensitivity and specificity in lymph node staging between the FDG and FAPI groups (*P* > 0.05)^95^. However, the median age of the patients in this study was relatively high (68 years), which might have led to a higher incidence of pathological abdominal conditions such as preceding surgery or co-existing infection, that could influence the tracer accumulation of both FAPI and FDG^95^.

The prognostic value of FAPI-PET in gastric cancer has been demonstrated by several authors. One study demonstrated that SUVmax and TBR of ^68^Ga-FAPI-04 correlated with clinical outcomes (T) and lymph nodal status (N), which suggested FAPI-PET is a prognostic surrogate^92^. Another study suggested that FAP expression of the primary tumor was significantly higher in patients who did not benefit from immune checkpoint blockade (ICB) therapy, making FAPI-PET a potential predictor of immunotherapy response^74^. The data suggest that high expression of FAP might indicate a poor prognosis in metastatic GC and FAPI-PET may serve as a non-invasive biomarker to select patients who are likely to benefit from the ICB therapy^74^.

Thus, FAPI-PET has a sensitivity advantage over FDG-PET for gastric cancer and may provide prognostic information as well, which should be evaluated in further studies.

### Pancreatic cancer

In pancreatic ductal adenocarcinoma (PDAC), FAP overexpression is found not only in tumor stroma but also in the PDAC cells and may be associated with metastatic spread and worse clinical outcome^12,24^.

**Fig. 4:**
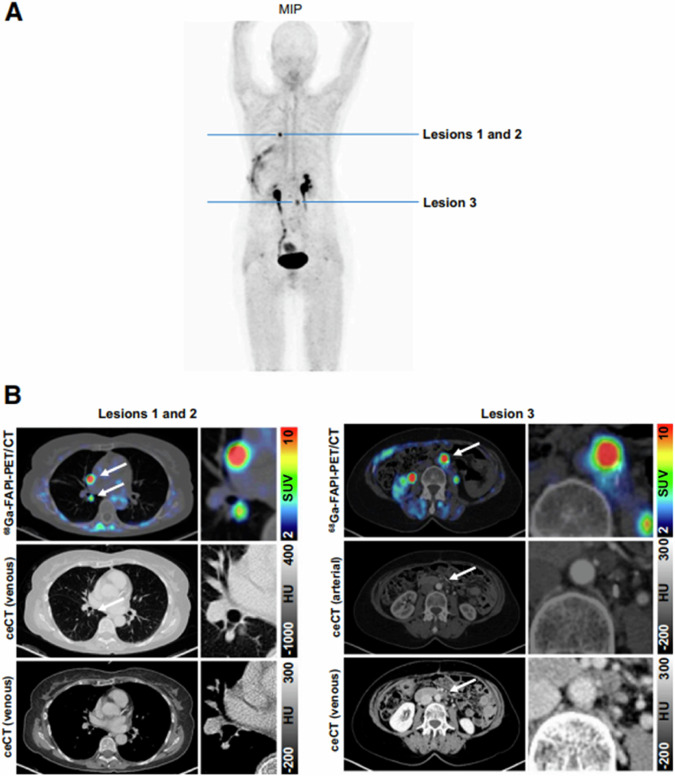
Example images of 64-year-old woman with recurrent pancreatic ductal adenocarcinoma. **A** Maximum-intensity projection (MIP) of ^68^Ga-FAPI PET. **B** Axial ^68^Ga-FAPI PET/CT images and contrast-enhanced CT (ceCT) images of lesions (arrows: lesions 1 and 2, pulmonary metastasis and mediastinal lymph node metastasis; lesion 3, paraaortic lymph node metastasis) detected by ^68^Ga-FAPI PET. HU: Hounsfield units. [From ref. ^99^].

FAPI-PET outperforms FDG-PET in sensitivity and detection rate in PDAC^96–102^. In one study, FAPI-PET was superior to FDG-PET in detecting the primary tumor (100% vs. 95.0%), metastatic lymph nodes (96.2% vs. 61.5%), and distant metastases (100% vs. 84.0%) (all *P* < 0.0001)^96,97^. Consequently, disease management was altered in many cases^96–98,101,102^. Other studies suggest that FAPI-PET can alter tumor stage in 43% compared with conventional imaging^99^ (Fig. 4).

In addition to detecting the extent of pancreatic cancer, FAPI-PET may also provide prognostic information^96,102,103^. For instance, one study established that tumors with ^68^Ga-DOTA-FAPI-04 PET/CT SUVmax > 14.9 before chemotherapy were more likely to progress^96^. Others have suggested that higher baseline metabolic tumor volumes (MTV) of ^18^F-AlF-NOTA-FAPI-04 were associated with poorer overall survival, although the hazard ratio was minimally elevated (hazard ratio HR = 1.016, *P* = 0.016)^102^. In treatment-naïve patients, ^68^Ga-FAPI-04 uptake correlates with both ex-vivo FAP expression and aggressive pathological features^103^. For instance, FAP expression was higher in poorly differentiated PDAC than in well- to moderately differentiated neoplasms^103^. Tumor uptake was also significantly correlated with tumor size, differentiation, and perineural invasion^103^. In this study, SUVmax was a significant independent prognostic predictor of recurrence-free survival (HR = 2.46, *P* < 0.05)^103^.

False positive results due to inflammatory conditions, such as pancreatitis, are an ongoing concern^98,104^. For the differentiation of inflammation from malignancy, the kinetics of uptake may be important. One group suggested that uptake values be obtained at 10 min, 1 h, and 3 h post-injection. Decreasing uptake was observed in pancreatitis (SUVmax 7.24, 6.55, and 5.63 after 10, 60, and 180 min, respectively), whereas the uptake of PDACs was stable or slightly increased (SUVmax 11.48, 12.66, and 13.23 after 10, 60, and 180 min, respectively)^98^. The earlier washout of FAPI-PET agents in inflammation was further confirmed in a separate study in patients with pancreatitis, where reduced SUVmax on 3 h delayed static scans were observed, while malignant lesions showed no significant tracer washout^104^.

FAPI-PET has a very promising role to play in PDAC diagnosis and staging. It is more sensitive than FDG-PET and with the use of washout kinetic studies at three hours, FAPI-PET may be able to distinguish benign and malignant lesions.

### Colorectal cancer

Much like other GI tract tumors, increased FAP expression is associated with higher tumor aggressiveness and poor prognosis in colorectal cancer (CRC)^13,14,105,106^. While the majority of CRCs are FDG avid, the diagnostic performance of FDG imaging in CRC is substantially limited by high physiological bowel uptake^41^. Accordingly, head-to-head comparisons of FAPI and FDG generally show superior performance in sensitivity, detection rate, TBR, and higher SUVmax for FAPI-PET.

Multiple studies have reported a higher sensitivity of FAPI-PET compared to FDG-PET for the detection of primary CRC tumors (100% vs. 53%)^107^, lymph node metastases (90% vs. 80%)^108^, distant metastases (89% vs. 57%), and peritoneal metastases (100% vs. 55%)^107,108^. The specificity of FAPI- vs. FDG-PET for nodal metastases was also higher (100% vs. 81.8%)^108^. This is especially true of specific subtypes of CRC such as signet-ring cancer that tends to be non-FDG avid^108–110^. TBR of most CRC lesions was significantly higher on FAPI-PET^108–110^. SUVmax of primary lesions, peritoneal, and liver metastases was higher than in FDG-PET^108,109^, except for lymph node metastases^107^. FAPI-PET led to an overall change of TNM stage in 50% of the treatment-naïve patients^108^ or led to a change of treatment options in 21% of patients with confirmed primary CRC^109,110^. Another study demonstrated that higher TBR was obtained from ^68^Ga-FAPI-04 vs. ^18^F-FDG-PET scans (13.3 vs. 8.2) in primary tumors^109^. Both SUVmax in peritoneal metastases and TBR in liver metastases of ^68^Ga-FAPI-04 were higher than those of ^18^F-FDG (5.2 vs. 3.8 and 3.7 vs. 1.9, both *P* < 0.001)^109^. Clinical TNM staging based on ^68^Ga-FAPI-04 PET/CT led to upstaging and downstaging in 16% and 8.2% respectively^109^.

### Peritoneal carcinomatosis

Peritoneal carcinomatosis occurs in multiple cancer types, especially in ovarian cancer, gastric, and colorectal cancer^111^. FDG has poor sensitivity for identifying peritoneal carcinomatosis, due to the physiological FDG uptake in the GI tract that masks carcinomatosis, and the low FDG avidity in some cancer subtypes^42^. Overall, FAPI-PET exhibits higher sensitivity and TBR^112^ for peritoneal carcinomatosis than FDG^90,108^.

Many studies confirm the higher sensitivity of FAPI-PET for peritoneal carcinomatosis. In colorectal carcinomatosis, ^68^Ga-FAPI-04 PET/CT exhibited a higher sensitivity (100% vs. 40%) with a comparable specificity to FDG (both 100%) for the detection of peritoneal involvement^108^, although not all studies reach the same conclusion^90^. In peritoneal gastric cancer, ^68^Ga-FAPI-04 had a sensitivity 100% vs. 40% for ^18^F-FDG, while both scans had a high specificity of 100%^90,92^. ^68^Ga-DOTA-FAPI-04 also showed higher TBR in peritoneal metastases (8.1 vs. 3.2, *P* < 0.001) compared to ^18^F-FDG-PET/CT^92^.

**Fig. 5:**
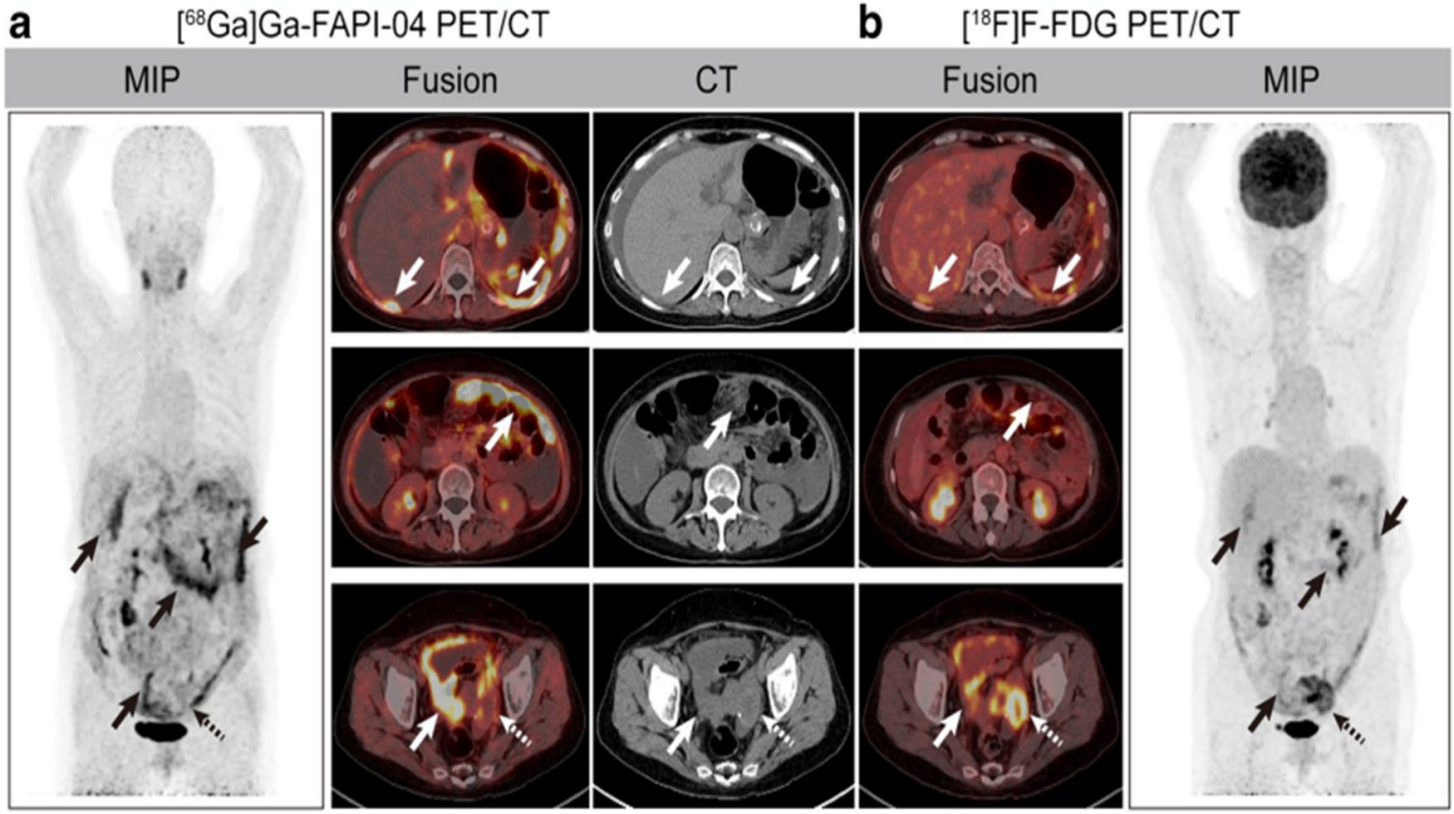
A 66-year-old woman underwent preoperative staging after being diagnosed with left ovarian high-grade serous carcinoma. ^68^Ga-FAPI-04 PET/CT demonstrated mild focal uptake (SUVmax, 4.5) in the left primary tumor ((**a**); dotted arrow head) and distinctive uptake (SUVmax, 9.7) in widespread peritoneal metastasis ((**a**); solid arrowhead). ^18^F-FDG PET/CT revealed high focal uptake (SUVmax, 8.8) in this cystic-solid ovary tumor ((**b**); dotted arrowhead) and slight and diffuse uptake (SUVmax, 2.5) in the peritoneal metastases ((**b**); solid arrowhead). For this representative participant, ^68^Ga-FAPI-04 PET/CT detected more metastatic lesions compared with ^18^F-FDG PET/CT regarding the peritoneal metastases. [From ref. ^113^].

In peritoneal ovarian cancer, FAPI-PET demonstrated superior sensitivity compared to FDG^112–114^. In one study, ^68^Ga-FAPI-04 PET/CT showed higher sensitivity for detecting peritoneal metastases (97% vs. 76%; *P* < 0.001)^113^ (Fig. 5) and higher SUVmax (17.31 vs. 13.68; *P* = 0.026), leading to upstaging in many patients^113^. TBRs for FAPI-PET were also consistently higher than FDG-PET (median TBR 5.8 vs. 2.7, respectively; *P* < 0.001)^112^. Thus, FAPI-PET, across a broad range of cell types, demonstrates substantially better sensitivity for peritoneal carcinomatosis with higher specificity compared to FDG-PET.

### Lung cancer

Since lung cancer (LC) is the most common malignancy, the performance of FAPI-PET compared to FDG-PET has generated multiple studies^112–116^. Overall, FAPI-PET surpasses FDG-PET for detecting primary cancer and metastases in the brain, pleura, bone, and lymph nodes^115–118^. In one study comparing patients with all forms of LC, ^18^F-FAPI-74 PET/CT demonstrated higher sensitivity (99% vs. 87%; *P* < 0.001), specificity (93% vs. 79%; *P* = 0.004), and accuracy (97% d vs. 85%; *P* < 0.001)^118^. In a similar study of ^68^Ga-FAPI, a higher detection rate of primary tumors and metastases including lymph nodes, brain, bone, and pleura was found when compared to ^18^F-FDG-PET/CT^117^. A standard limitation of such studies is that histologic confirmation is usually not possible and confirmation often relies on other methods such as conventional imaging and clinical history^117^.

Several subtypes of LC are less avid for FDG-PET and therefore, might be expected to yield even better results for FAPI-PET. For instance, in patients with lung adenocarcinoma ^18^F-AlF-NOTA-FAPI-42 PET/CT outperformed ^18^F-FDG, demonstrating higher SUVmax in lymph nodes, pleura, bones, and other distant metastases^115^. Moreover, ^18^F-FAPI-PET detected more lesions than ^18^F-FDG (total lesions 554 vs.464, *P* = 0.003), lymph nodes (258 vs. 229, *P* = 0.039), brain metastases (34 vs. 9, *P* = 0.002), and pleural metastases (56 vs. 30, *P* = 0.041)^115^. A comparison between different subtypes of LC including adenocarcinoma (ADC), squamous cell carcinoma (SCC), small cell lung cancer (SCLC) revealed no difference between ^18^F-AlF-NOTA-FAPI-04 and ^18^F-FDG^119^ with the minor exception that bone metastases of SCC and ADC exhibited higher uptake than those of SCLC^119^.

False positive uptake is a limitation of FAPI-PET in staging lung cancer. Various benign conditions e.g., co-existing post-radiation injury, surgery, or inflammation may also lead to positive FAP accumulation, thus making the precise differentiation between malignant and benign pulmonary findings challenging^120–127^. Inflammatory lesions tend to exhibit statistically lower uptake compared to cancers. Despite statistically different results among the groups, the degree of overlap between the two categories makes distinguishing benign and malignant disease in a specific patient, problematic^128^. The role of tracer washout in distinguishing inflammation from cancer is yet to be investigated. Thus, consensus has not yet been reached on the utility of FAPI-PET *vis a vis* FDG-PET in lung cancer^128^.

### Breast cancer

The reported presence of abundant stroma in most primary breast cancers^17,129^ and the reported FAP expression in breast cancer cells^16^ suggest a highly promising role for FAPI-PET. FDG-PET has known limitations in some forms of breast cancer, particularly in invasive lobular breast cancers (ILC), which are characterized by lower tumor cell density^17^ and lower FDG uptake^130,131^. In this setting, FAPI-PET may provide a valuable alternative for diagnosis and staging, although specific analysis of different histological types is still lacking.

Numerous studies have demonstrated the effectiveness of FAPI-PET in detecting primary and metastatic breast cancer lesions with high sensitivity and specificity^132^. ^68^Ga-FAPI-04 PET/CT was superior to ^18^F-FDG PET/CT in detecting primary tumors demonstrating higher sensitivity, SUVmax, and TBR^133^. Additionally, ^68^Ga-FAPI-04 PET/CT was superior to ^18^F-FDG PET/CT in detecting lymph nodes, hepatic, bone, and cerebral metastases^133^. ^68^Ga-FAPI PET/CT also detected a higher number of primary breast lesions due to higher tracer uptake compared to ^18^FDG-PET/CT^134^. In one study, FAPI was effective in the major histologic subtypes (invasive ductal carcinoma: IDC 89.6%) and ILC (10.4%), while ^18^F-FDG showed lower uptake, especially in the ILC subtype^134^. In a selected cohort of patients with low FDG uptake, FAPI-PET demonstrated higher sensitivity and uptake^135^. In another study, FAPI-PET consistently demonstrated higher SUVmax (11.06 ± 5.48 vs. 8.33 ± 6.07, *P* = 0.02) and TBR (15.32 ± 10.33 vs. 8.25 ± 5.51, *P* < 0.001) compared to ^18^F-FDG in primary tumors, confirming the results of previous studies^136^.

Despite these promising results, there are a number of limitations of FAPI-PET in the diagnostic imaging of breast cancer. Hormonal status can influence uptake with pre-menopausal patients, showing higher uptake in the normal breast than post-menopausal women. Uptake is significantly increased with lactation^137^. A higher background in pre-menopausal women can reduce TBR and consequently reduce accuracy^137^. Additional limitations of FAPI-PET, which may potentially compromise diagnostic accuracy will be discussed in the “Limitations” section.

### Ovarian cancer

FAP is expressed in most primary epithelial ovarian cancers with most of the expression arising from the tumor stroma, but some arising from the tumor cells themselves^138^. Mhawech-Fauceglia et al. evaluated ex vivo the expression of FAP in 338 primary epithelial ovarian cancer specimens and found positive FAP expression in 70 cases (21%) in neoplastic cells and 207 cases (61%) in the tumor stroma^138^. Notably, almost all cases (66/70), which expressed FAP in the neoplastic cells also expressed FAP in the stroma, while 42% showed FAP expression only in the stroma^138^. Since FAP expression in ovarian cancers was shown to be associated with cancer promotion, invasion, chemoresistance, and worse clinical outcomes, it is not surprising that FAP theranostics have been suggested as potential adjuvants to existing treatment paradigms^20,138,139^.

There have been several head-to-head comparative studies of FAPI- vs. FDG-PET in ovarian cancer^112–114,140^. Overall, FAPI-PET has demonstrated higher sensitivity for ovarian cancer compared to FDG-PET, especially in the detection of primary tumors, nodal, and distant metastases including peritoneal involvement and peridiaphragmatic metastases^114^. Although the SUVmax was comparable to FDG in some studies^112,140^, FAPI-PET scans generally showed higher TBR and higher detection rates of disease. For example, FAPI demonstrated improved sensitivity for detecting peritoneal metastases (96.8% vs. 83.0%), retroperitoneal (99.5% vs. 91.4%), and supradiaphragmatic lymph node metastases (100% vs. 80.4%) (all *P* < 0.001)^113^. ^68^Ga-FAPI-04 also showed higher SUVmax for peritoneal metastases (17.31 vs. 13.68; *P* = 0.026), retroperitoneal (8.72 vs. 6.56; *P* < 0.001) and supradiaphragmatic lymph node metastases (6.39 vs. 4.20; *P* < 0.001) compared to ^18^F-FDG^113^.

The hormone status of the patient influences background activity in the uterus and breasts^137^. There is significantly higher SUVmax in the endometrium (11.7 vs. 3.0; *P* < 0.001) and breast (1.8 vs. 1.0; *P* = 0.004) of pre-menopausal women than in post-menopausal women^137^. In contrast, FAPI accumulation in the ovaries showed no statistically significant differences between pre- and post-menopausal women (SUVmax 2.8 vs 1.6; *p* = 0.14)^137^. Overall, FAPI uptake in 167 female probands showed a mean SUVmax of 4.0 (±3.2) in endometrium (*n* = 128), 1.7 (±0.8) in ovary (*n* = 64), and 1.1 (±0.5) in breast tissue (*n* = 147)^137^. Although future validation is needed, lower physiological FAP accumulation in ovaries both in pre- and post-menopausal women, underscores the diagnostic potential of FAPI-PET in ovarian cancer.

### Sarcoma

While undifferentiated sarcoma and rhabdomyosarcoma are strongly FDG avid^141,142^, low-grade sarcomas such as myxoinflammatory fibroblastic sarcoma, low-grade leiomyosarcoma, low-grade liposarcoma, solitary fibrous tumor, myxoid liposarcoma, and synovial sarcoma are known to be less FDG avid and thus, it is more likely that FAPI-PET will be advantageous^141,143^.

The expression of FAP in sarcoma was recently evaluated ex vivo by Crane et al., who demonstrated that 78% of stromal cells and 51% of tumor cells exhibited positive FAP staining^144^. The highest stromal FAP expression was observed in desmoid fibromatosis, myxofibrosarcoma, solitary fibrous tumor, and undifferentiated pleomorphic sarcoma^144^. The FAP expression in sarcoma cells was initially described by Rettig et al. in 1988, who evaluated the binding of FAP antibody (F19) in 12 known sarcoma cell lines. The study found FAP expression in the cell lines of fibrosarcoma, malignant fibrous histiocytoma (MFH), leiomyosarcoma, osteosarcoma, chondrosarcoma, liposarcoma, synovial sarcoma, and undifferentiated sarcoma, although the number of samples in each category was small (*n* ≤ 10)^3^. Conversely, no FAP expression was found in embryonal rhabdomyosarcoma, Ewing sarcoma, mesenchymal chondrosarcoma, and rhabdomyosarcoma, again with small numbers in each category^3^. In subsequent studies, these early findings appear to have been confirmed^17,23^.

Clinical studies have shown a significant correlation between FAPI-PET uptake and histopathologic FAP expression^145^. The positive predictive value and sensitivity were 100% and 96%, respectively. However, detection rates of ^68^Ga-FAPI and ^18^F-FDG were comparable in this cohort with no clear advantage for either PET agent. The subgroup analysis was limited due to the small number of patients in each sarcoma subtype^145^. In another study, FAPI-PET/CT detected more lesions compared to FDG-PET/CT and outperformed FDG significantly in sensitivity, specificity, positive and negative predictive values, and accuracy (*P* < 0.001)^146^. Moreover, ^68^Ga-DOTA-FAPI-04 demonstrated significantly higher values of SUVmax and TBR compared to ^18^F-FDG-PET/CT in liposarcoma, malignant solitary fibrous tumor (MSFT), and interdigitating dendritic cell sarcoma (IDCS)^146^. However, mean SUVmax and TBR suggested that ^18^F-FDG was more sensitive than ^68^Ga-DOTA-FAPI-04 in undifferentiated pleomorphic sarcoma (UPS) (*P* = 0.003 and *P* < 0.001, respectively) and rhabdomyosarcoma (RMS) (*P* < 0.001)^146^. This finding agrees with Koerber et al., who reported high SUVmax and TBR in a cohort of 15 patients with various sarcomas^147^. The highest uptake was found in liposarcomas and high-grade disease. Moreover, a high SUVmax (>10) was observed for more aggressive disease, indicating the higher uptake of FAPI correlates with a higher grade of malignancy^147^.

Thus, a general statement regarding the efficacy of FAPI-PET cannot be made due to the variable nature of tumors that fall under the “sarcoma” rubric. Among sarcomas, undifferentiated pleomorphic sarcoma, high-grade osteosarcoma, high-grade liposarcomas, and the rare solitary fibrous tumors (SFT) are currently the sarcoma subtypes with the highest likelihood of FAPI uptake, which should be validated in a future study.

## Non-oncological indications

### Overview

**Fig. 6:**
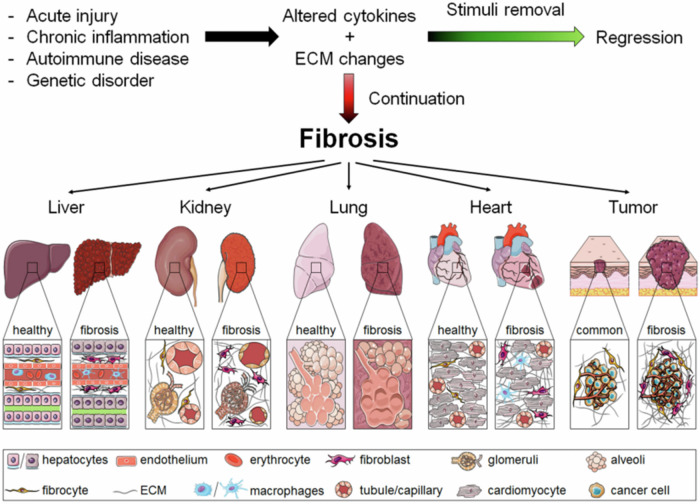
Pathological characteristics of fibrosis in different tissues. In tissues such as liver, kidney, lung, heart, and tumor, common events lead to fibrosis progression (and regression). If the pathological stimulus is persistent and the healing process is dysregulated, the continuous recruitment and activation of inflammatory cells and myofibroblasts can result in fibrosis. Core features of fibrotic processes that are shared by all of these organs include overproduction of cytokines, growth factors, ECM proteins, and ultimately the loss of tissue architecture as well as function. [From ref. ^148^].

Multiple benign processes result in fibroblast activation, e.g., fibrosis, inflammation, benign tumors, or scar formation which may be amenable to imaging with FAPI-PET^148^ (Fig. 6). It was observed that in many FAPI-PET studies obtained for cancer evaluation, non-malignant findings were very common^149^.

The mechanisms by which fibroblasts are activated are multifaceted, complex, and beyond the scope of this review. The most common pathway is thought to arise from transforming growth factor (TGF)-β signaling^149–153^. TGF-β modulates multiple cellular processes including synthesis of ECM^150,154–156^. and regulates the differentiation of fibroblasts into myofibroblasts^157^, thus playing an essential role in initiating and sustaining fibrosis^150–152^. Additionally, TGF-β takes part in the pathological wound-healing process and is considered to play an essential role in tissue inflammation^158–161^, suggesting a close relationship between inflammatory and fibrotic processes.

### Myocardial infarction

Not surprisingly, myocardial infarction demonstrates FAPI uptake due to ischemia-induced fibroblast activation^162^. In a preclinical study, it has been shown that after the left coronary artery was ligated, ^68^Ga-FAPI-04 uptake in the injured myocardium peaked on day 6^162^. The tracer accumulation in the myocardial corresponded with the region of decreased ^18^F-FDG uptake^162^. Histopathologic evaluation revealed that ^68^Ga-FAPI-04 accumulated mainly at the border zone of the infarcted myocardium, suggesting this area is the most fibrotically active due to ongoing tissue remodeling and reparatory processes in response to acute ischemia^162^.

**Fig. 7:**
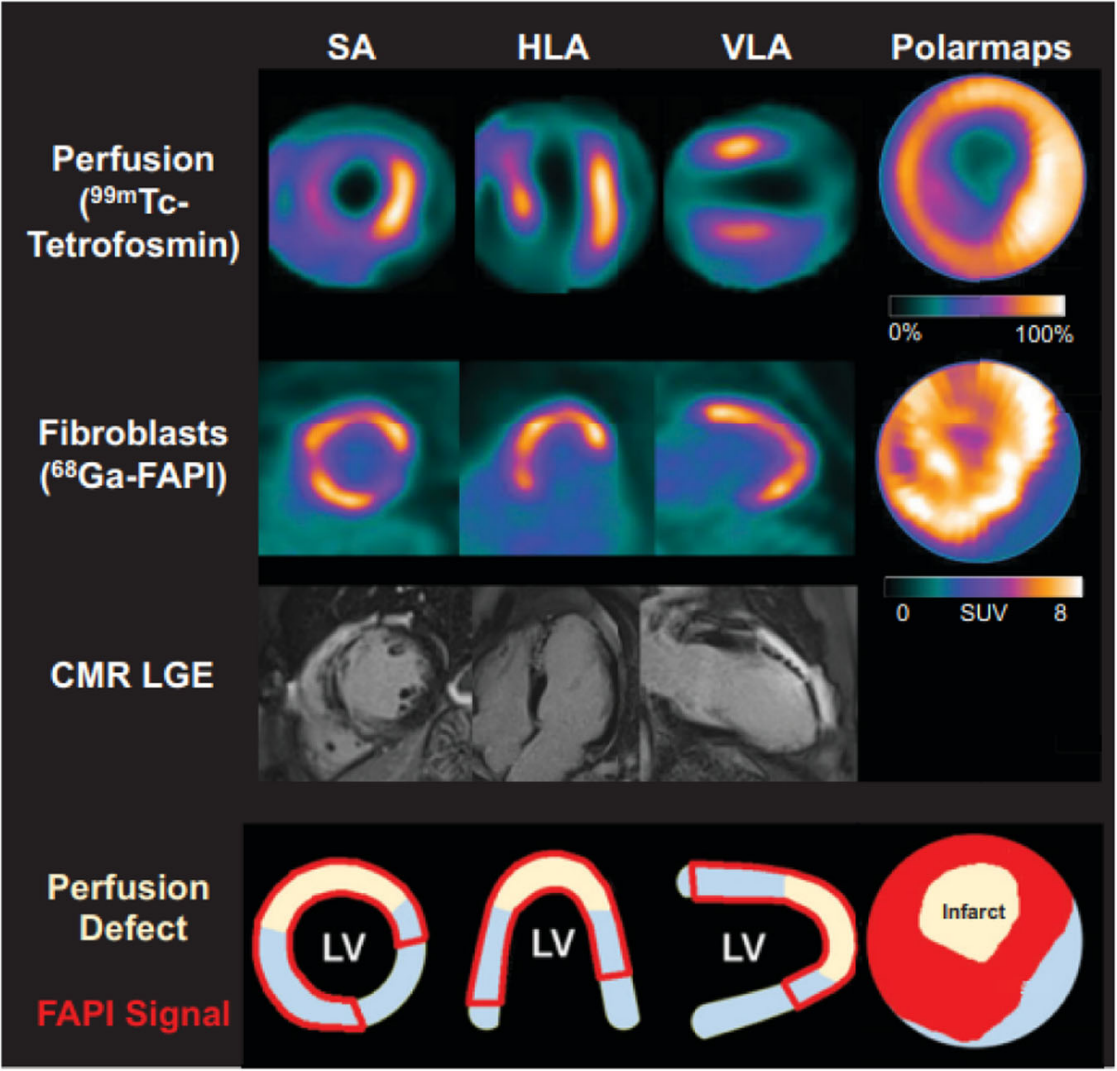
Myocardial perfusion images using 99mTc-tetrofosmin at rest, ^68^Ga-FAPI PET, LGE from CMR, and schematic drawings of LV. Area of fibroblast activation as indicated by ^68^Ga-FAPI-46 PET signal exceeds infarct area and LGE signal, the most common type of myocardial FAP distribution. HLA 5 horizontal long axis; SA 5 short axis; VLA 5 vertical long axis. [From ref. ^163^].

In humans, Diekmann et al. demonstrated the prognostic significance of early cardiac fibroblast activation after the acute myocardium infarction (AMI)^163^. In this study, 35 patients underwent cardiac MRI, perfusion SPECT, and ^68^Ga-FAPI-46 PET/CT after AMI (^68^Ga-FAPI-46 PET/CT was performed on day 7.5 (±1.3 d) after AMI)^163^. The FAP-positive region was significantly larger than the area of SPECT perfusion defect (*P* < 0.001) or the infarct area evaluated by cardiac MRI (*P* < 0.001)^163^ (Fig. 7). These areas include viable tissue in the border zone, where the injury may contribute to the development of interstitial fibrosis^163^. The FAPI-PET-based myocardial injury volume was predictive of outcome after AMI^163,164^. Additionally, the PET signal was predictive of subsequent development of left ventricular (LV) dysfunction at follow-up (*r* = −0.58, *P* = 0.007)^114^. These results suggest that FAPI-PET might be a useful imaging tool in the setting of AMI, and serve as a predictor of ventricular remodeling after MI^163^. This conclusion is further supported by a study that assessed the predictive value of ^68^Ga-DOTA-FAPI-04-PET/MR for late LV remodeling after AMI^165^. FAPI uptake volume at baseline was a significant predictor (OR = 1.048, *P* = 0.011) for LV remodeling at 12 months after myocardial infarction^165,166^.

### Cardiac fibrosis

Approximately, 15–20% of cells in the adult heart are identified as cardiac fibroblasts^163^. Cardiac damage triggers fibroblast activation^167^, leading to adverse remodeling of cardiac tissue, characterized by replacement or reactive fibrosis^168^. Therefore, activated cardiac fibroblasts might serve as an attractive therapeutic target to modulate the remodeling process and improve functional outcomes^169,170^. Some preclinical studies using mouse models of cardiac fibrosis suggest the possibility of selectively targeting activated fibroblasts to arrest or even reverse reactive fibrosis^171–173^. In a pressure overload heart failure model in Sprague-Dawley rats, FAPI uptake both in the heart and liver correlated with the early stages of fibrosis^174^, confirming the well-known connection between chronic heart failure and congestive liver fibrosis^175^. FAPI uptake in the heart muscle also correlated with right ventricular pressure overload, leading to cardiac hypertrophy and fibrosis after pulmonary artery banding^176^.

Cardiac fibrosis is currently difficult to assess and FAPI-PET may thus, be a welcome tool to assist cardiologists^177,178^. In a study of hypertrophic cardiomyopathy (HCM), FAPI-PET demonstrated intense and inhomogeneous myocardial uptake across the LV myocardium (median target-to-background ratio, 8.8 vs. 2.1 in healthy controls; *P* < 0.001)^179^. In HCM, more segments with elevated FAPI uptake were detected than the number of hypertrophic segments (median; 14 vs. 5; P < 0.001), possibly indicating functionally active regions of disease^179^. The degree of FAPI accumulation was shown to correlate with the 5-year sudden cardiac death (SCD) risk score (*r* = 0.32; *P* = 0.03)^179^. FAPI uptake in the left ventricle was also associated with obesity, diabetes mellitus, and radiation exposure to the chest^180^. However, the possible prognostic value of FAPI-PET in cardiac fibrosis will require further research before it is used as a routine clinical tool.

### Liver fibrosis

Liver fibrosis is a precursor of liver malignancies and hepatic dysfunction, with 80%–90% of hepatic malignancies developing in fibrotic or cirrhotic liver parenchyma^181^. Activated hepatic stellate cells are believed to be the source of hepatic fibrosis and are known to express FAP, thereby promoting fibrosis in the liver^182^. A quantitative, non-invasive measure of fibrosis would be useful clinically. In a swine model, hepatic fibrosis as measured by the collagen proportionate area (CPA) correlated with ^68^Ga-FAPI-46 uptake (*r* = 0.89, *P* < 0.001)^183^. ^68^Ga-FAPI-46 uptake increased progressively with increasing hepatic fibrosis^183^. The strong correlation between liver ^68^Ga-FAPI-46 uptake and the histologic stage of liver fibrosis suggests that ^68^Ga-FAPI-PET can play an impactful role in non-invasive staging of liver fibrosis^183^.

Because tumors arise in a background of fibrosis, theoretically, this could interfere with the detection of cancer^77^, on the other hand, simply identifying sites of significant fibrosis could help facilitate the early detection of liver malignancies. In actual practice, SUVmax and lesion-to-background ratio (LBR) of ^18^F-FAPI-74 PET were significantly higher in hepatocellular carcinoma than in benign fibrosis (HCC: SUVmax: 6.4 vs. 4.5, *P* = 0.017; LBR: 5.1 vs. 1.5, *P* = 0.003)^184^. These findings suggest that FAPI-PET imaging could differentiate malignant from non-malignant and non-inflammatory fibrosis in the liver, potentially making it a useful screening test in high-risk individuals.

### Lung fibrosis/inflammation

Pulmonary fibrosis is a common disorder and is largely detected by pulmonary function testing followed by computed tomography. Unchecked, it can lead to severe disability and death, however, early intervention can prevent many later sequelae. Interstitial lung diseases (ILD) can arise from multiple causes, e.g., idiopathic pulmonary fibrosis (IPF), systemic sclerosis, or radiation among others^185–188^.

Pulmonary fibrosis is usually the result of acute lung inflammation that fails to resolve over time, causing the deposition of fibrosis in the lungs^150,161,185^. Thus, acute inflammation can lead to chronic inflammation and finally, to fibrosis, a pattern replicated in many organs^150^. Once it begins, pulmonary fibrosis can destroy lung architecture and lead to respiratory failure^150,161^. It is thought that wound healing dysregulation is the initiating step in fibrosis development^150,158^. Myofibroblasts are one of the key cells in wound healing but if they fail to regress after healing can eventually cause pulmonary fibrosis^150,189^. The differentiation of fibroblasts into myofibroblasts is regulated by the secretion of TGF-β and mechanical stress^150,190^. Myofibroblasts actively synthesize ECM components during lung tissue repair. During chronic inflammation, myofibroblasts evade apoptosis, forming hyperproduction of ECM and finally, pulmonary fibrosis^150,190^. In vitro data suggest that FAP expression is significantly increased, even in the early phase of profibrosis^191^.

FAPI-PET/CT uptake in patients with systemic sclerosis-associated ILD was higher in patients with progressive disease, compared to those with stable or inactive disease^192^. This study also found that increased ^68^Ga-FAPI-04 uptake at baseline was associated with ILD progression within 6–10 months, independently of the extent of involvement on initial high-resolution CT (HRCT) scan and the forced vital capacity at baseline^192^. The potential predictive value of FAPI-PET has been demonstrated in several studies^191,193,194^. In one study of ILD, baseline SUVtotal of ^68^Ga-FAPI-04 was significantly related to the pulmonary functional decline (decrease of vital capacity) (*r* = −0.5257, *P* = 0.0017)^191^.

Unfortunately, pulmonary inflammation alone or in combination with ILD can lead to FAPI-PET uptake^191^. Thus, increased activity in the lung can be due to inflammation or fibrosis, and teasing out the component of each can be challenging^124,126,195,127^. For example, patients with SARS-CoV-2 infection and suspected pulmonary fibrosis, have increased uptake of ^68^Ga-FAPI-46-PET/CT compared to the control group^196^. However, it remains unclear, whether such uptake represents inflammation, or early fibrotic change, not visible on CT. Inflammation and fibrosis share a common TGF-β activation pathway and distinguishing between the two entities is predicted to be difficult.

### Kidney fibrosis

The final common pathway of chronic kidney disease (CKD) is renal fibrosis. The severity of fibrosis correlates with the degree of renal dysfunction and its reversibility. In preclinical studies, high FAP expression was observed in animals with CKD and the expression increased with the progression of renal fibrosis. In another preclinical study, higher SUVmax and TBR in ^68^Ga-FAPI-04-PET/CT were found in animals with CKD, but not in controls^197^. These results indicate the potential usefulness of FAPI-PET for non-invasive evaluation of renal fibrosis with the potential to reduce the need for renal biopsy^197^.

In a clinical study, three different radiotracers, ^68^Ga-FAPI, ^68^Ga-PSMA, and ^68^Ga-DOTATOC were compared in CKD patients^198^. The authors found a negative correlation between the glomerular filtration rate (GFR) and ^68^Ga-FAPI uptake, indicating the functional decline of kidney is associated with higher FAPI accumulation in the parenchyma^198^. Meanwhile, neither ^68^Ga-DOTATOC nor ^68^Ga-PSMA uptake correlated with CKD stage^198^. In another study in patients with confirmed renal fibrosis, ^68^Ga-FAPI-04-PET/CT SUVmax measurements in mild, moderate, and severe fibrosis were 3.92 ± 1.50, 5.98 ± 1.6, and 7.67 ± 2.23, respectively, demonstrating an increase in uptake with increased fibrosis^199^.

Overall, FAPI-PET may be a reasonable surrogate of renal fibrosis and therefore, may be able to predict the severity of fibrosis, monitor response to anti-fibrotic therapies, and help determine patient outcomes.

### Rheumatoid arthritis

Rheumatoid arthritis, along with other auto-immune arthritides, are systemic illnesses that are often treated with systemic therapies. While the mainstay of assessment is self-reported patient symptoms, imaging can play an important role in the quantitative assessment of disease burden and response to therapy. Fibroblast-like synoviocyte cells (FLSs) are central to the formation of joint inflammation and are known to overexpress FAP^200^.

**Fig. 8:**
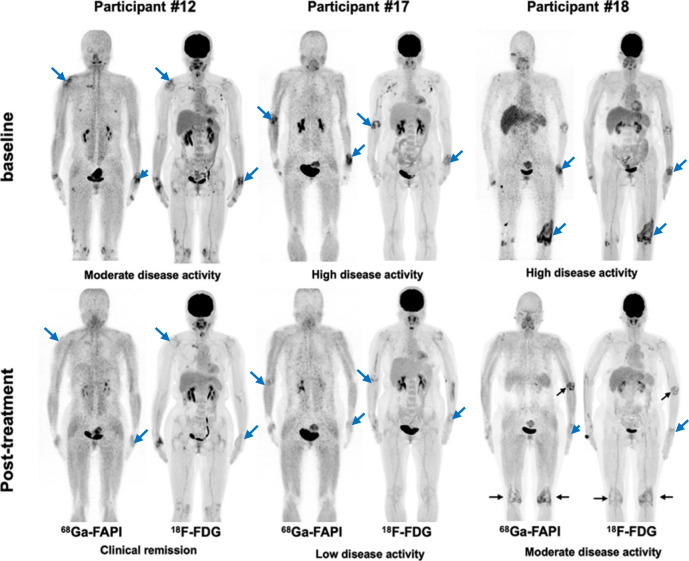
Pre- and post-treatment dual-tracer PET/CT in the three participants with different responses undergoing tight control treatment. Participant 12 is a 55-year-old woman with a 1-month history of rheumatoid arthritis (RA) who was treated with methotrexate, etoricoxib, tripterygium wilfordii, and iguratimod. Participant 17 is a 53-year-old woman with a 1-year history of RA who was treated with methotrexate and etanercept. Participant 18 is a 55-year-old woman with a 19-month history of RA who was treated with methotrexate, etanercept, and tripterygium wilfordii. There is residual active uptake of ^68^Ga-FAPI and ^18^F-FDG in three major joints 6 months after treatment in participant 18 (black arrows). Major joints with rheumatoid affection are marked with blue arrows. [Adapted, from ref. ^202^].

Preclinical models of rheumatoid arthritis (RA), including the collagen-induced arthritis (CIA) mouse model, have demonstrated that the activated FLSs are detectable with FAPI-PET^200,201^. For instance, ^18^F-FAPI-04 uptake has been shown to correlate with inflammation and response to treatment^201^. In a clinical study comparing ^18^F-FDG-PET with ^18^F-AlF-NOTA-FAPI-04, the latter demonstrated higher uptake in inflamed joints even at an early stage of arthritis and showed a positive correlation with clinical arthritis scores (*r* = 0.834, *P* < 0.001)^200^. ^68^Ga-FAPI-04 has substantially higher sensitivity for affected joints than ^18^F-FDG-PET/CT imaging^202^. The SUVmax value of the most affected joint in each participant was higher with ^68^Ga-FAPI compared to ^18^F-FDG PET/CT (9.54 vs. 5.85; *P* = 0.001)^202^. The ^68^Ga-FAPI-04 uptake in the joints correlated significantly with the clinical and radiographic grade of joint damage^202^, showing the therapeutic effect after anti-rheumatic treatment (Fig. 8).

These results suggest that FAPI-PET may be a promising radiotracer for aiding diagnosis and monitoring disease progression or therapy response, especially in difficult cases such as seronegative rheumatoid arthritis, which exhibits no classical immunological markers and can be difficult to diagnose and monitor^203^.

### IgG4-related disease

Immunoglobulin G4-related disease (IgG4-RD) is a relatively recently identified systemic fibroinflammatory disease, characterized by lymphoplasmacytic infiltration and variable degrees of fibrosis^204^. The disease can occur in virtually any organ, but most commonly is found in lymph nodes, liver, pancreas, retroperitoneum, lacrimal, and salivary glands^205,206^.

Here again, ^68^Ga-FAPI-PET/CT is more sensitive for organ involvement, particularly in the pancreas, bile duct, liver, and salivary glands, than FDG-PET^204^. However, FDG avid lymph nodes did not accumulate ^68^Ga-FAPI, which was attributed to the fact that these lymph nodes lacked the characteristic storiform fibrosis^207^, which is one of the defining histopathologic features of IgG4-RD^208,209^. As with other similar diseases, a distinction between inflammation and fibrosis remains important, as the latter is less likely to be reversible. One study showed the possibility of distinguishing inflammatory from fibrotic activities, demonstrating that ^68^Ga-FAPI-04-PET uptake in IgG4-RD was associated with abundant FAP-positive activated fibroblasts, while ^18^F-FDG-PET positive lesions showed dense lymphoplasmacytic infiltration of IgG4 positive cells^210^.

Therefore, FAPI-PET may be useful in assessing IgG4-RD organ involvement and is likely more sensitive and specific than FDG-PET.

### Crohn´s disease

Crohn’s disease (CD) is a chronic disease that causes inflammation throughout the digestive tract, particularly in the small and large bowel. Although fibrosis and inflammation are hallmarks of CD and differentiating between them has clinical relevance for treatment, current imaging modalities are not predictive^211,212^.

MR and CT enterography have become standard imaging methods for assessing bowel narrowing and fistula formation in CD^212^. FAPI-PET/MR enterography is a promising diagnostic tool for differentiating bowel wall fibrosis and inflammation^212^. In addition to showing anatomic regions of bowel lumen narrowing on MRI, SUVmax of FAPI-PET was significantly higher in segments with fibrosis compared to inflammation (7.6 vs. 2.0; *P* < 0.001)^212^. In severe fibrosis, SUVmax was higher than in mild to moderate fibrosis (8.9 vs. 6.2; *P* = 0.045)^212^. In addition, bowel segments with isolated active inflammation had lower FAPI uptake than segments with combined active inflammation and fibrosis (SUVmax, 3.2 vs. 8.1; *P* = 0.005)^212^. The sensitivity and specificity of FAPI-PET/MR enterography were 93% and 83%, respectively^212^. In another study^211^, FAPI-PET/CT showed superior performance compared to CT enterography (CTE) with a higher sensitivity for involved bowel segments: 93.3% vs. 86.7%)^211^. ^68^Ga-FAPI-04 PET/CT correlated well with endoscopy, CTE, and clinical biomarkers of CD^211^. Higher signal-to-background ratios were associated with clinical severity determined by the CTE score (*r* = 0.81; *P* < 0.0001)^211^. Further, the global FAPI-PET/CT score (defined as the sum of TBR in the affected segments, divided by the number of studied intestinal segments^213^) correlated with biomarkers such as C-reactive protein (CRP) or clinical indices for disease severity such as Crohn’s disease activity index (CDAI) or Crohn’s disease endoscopy index of severity (CDEIS). These findings indicate that FAPI-PET/CT might be a reliable surrogate for non-invasive assessment of disease activity in CD^211^. Further, FAPI-PET may also be useful in differentiating Crohn’s disease from ulcerative colitis (UC)^214^, because FAPI uptake was strongly increased only in CD but not in UC^214^. This result supports other in vitro studies that demonstrate the overexpression of FAP is found in the intestinal myofibroblasts in CD, but not in the corresponding areas of UC^215^.

### Erdheim–Chester disease

Erdheim–Chester disease (ECD) is a rare disease characterized by the abnormal accumulation of histiocytes, or tissue macrophages. ECD involves multiple organs and tissues and has diverse manifestations, which makes it difficult to distinguish ECD from other diseases^216^.

ECD is characterized by varying degrees of organ fibrosis^217^. The most common manifestations of ECD are in the bone, heart, brain, skin, lung, kidney, peritoneum, or omentum^218^. ^68^Ga-FAPI-PET/CT is more sensitive than ^18^F-FDG-PET/CT for detecting lesions^218^. Moreover, the SUVmax for ^68^Ga-FAPI-PET/CT was significantly higher than ^18^F-FDG-PET/CT in the heart (4.9 vs. 2.8; *P* = 0.050), lung or pleura (6.8 vs. 3.1; *P* = 0.025), peritoneum or omentum (5.7 vs. 2.8 ± 1.7; *P* = 0.032), and kidney or perinephric infiltration (4.9 vs. 2.9; *P* = 0.009)^218^. The utility of FAPI-PET in ECD is still limited and further work is needed to better understand its role in the management of these patients.

## Limitations

**Fig. 9:**
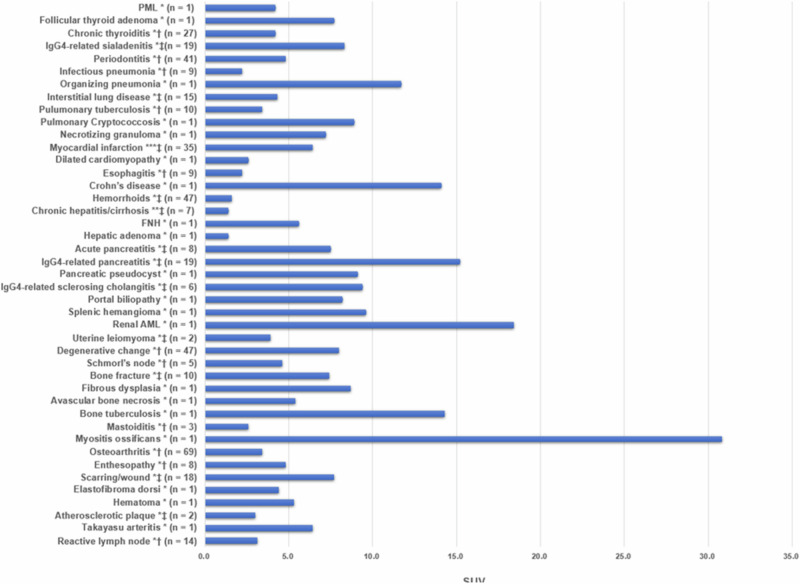
FAP tracer uptake in various non-oncologic diseases. Reported SUVs of non-oncologic diseases (*SUVmax, **SUVmean, ***SUVpeak, † median, ‡ average). SUV standardized uptake value. [From ref. ^120^].

While we have focused on some of the opportunities afforded by FAPI-PET, there are also several inherent limitations^44,120,219^. CAFs are intrinsically heterogeneous in origin, phenotype, and function^68^, which impact the amount of FAP expression. The heterogeneity of FAP expression, especially in non-oncological conditions may lead to false positive findings, as reported in numerous studies and case reports. Most of the false positives are associated with fibrous or sclerotic foci, including post-radiation injury or scar formation, causing activation of quiescent fibroblasts^149,220^. In a systematic search for studies with non-malignant FAPI PET/CT findings, a total of 1178 papers comprising a total of 2372 FAPI avid non-malignant findings were reported, with wide-ranging spectrum of benign diseases^149^ including inflammation. The most frequent incidental finding was the uptake of FAPI-PET in atherosclerosis, followed by degenerative and traumatic bone and joint disease or arthritis^149^. FAPI avid lymph nodes and tuberculosis are potential false positives in cancer staging^149^. These findings and their respective detection thresholds are depicted in Fig. 9.

It may also be difficult to distinguish between malignant and benign conditions on FAPI-PET. For instance, previous extended surgery near the region of interest may lead to false positive results for recurrence due to wound healing. In ovarian cancer patients, postoperative findings in the omentum and in the abdominal wall (at the surgical incision site) may also be a source of false positives^140^. Upon histologic evaluation, these areas are typically characterized microscopically by fibrous tissue hyperplasia and calcium salt deposition, that can cause false positive findings on FAPI-PET^140^.

**Fig. 10:**
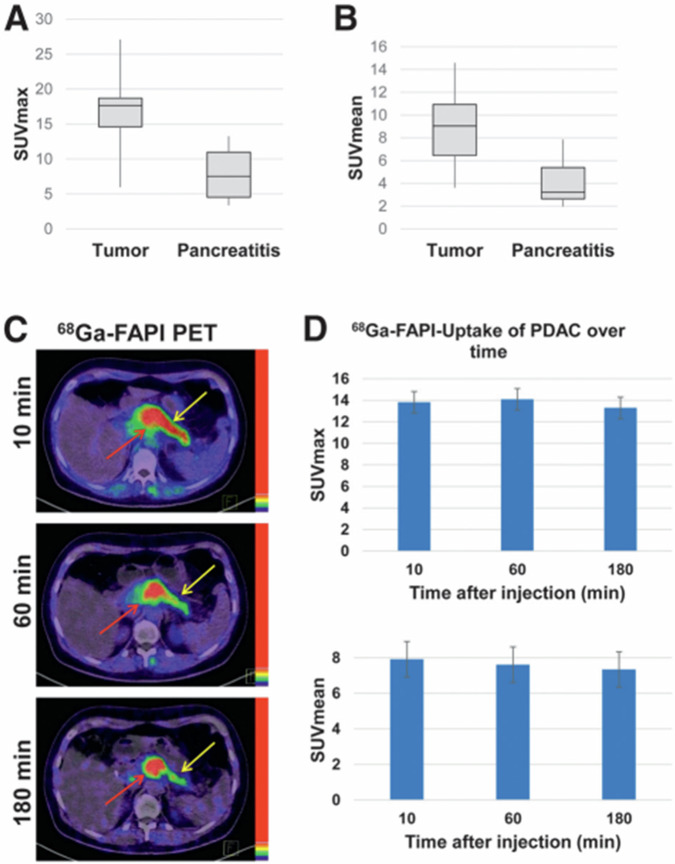
^68^Ga-FAPI tracer uptake in PDAC and in accompaniying pancreatitis. **A**, **B** Average SUVmax and SUVmean 1 h after injection of ^68^Ga-labeled FAPI tracers in 8 PDAC and in accompanying pancreatitis in rest of pancreas. **C** Exemplary images of tumor-related (red arrow) and pancreatitis-related (yellow arrow) ^68^Ga-FAPI uptake 10, 60, and 180 min after application. **D** ^68^Ga-FAPI uptake 10, 60, and 180 min after application (SUVmax and SUVmean values) in PDAC lesions of 6 patients. [From ref. ^98^].

Some investigators have suggested that the use of delayed static scans can differentiate inflammation from malignancy in the pancreas^98^. In pancreatitis, ^68^Ga-FAPI-PET/CT scan 3 h p.i. showed a significant decrease in uptake, while the uptake in pancreatic ductal adenocarcinoma was stable or slightly increasing^98,104,221^ (Fig. 10), indicating that this might provide a valuable, practical tool to non-invasively distinguish inflammatory vs. malignant conditions. Whether this method will prove useful in in other situations and whether such additional scans can be integrated into the clinical workflow without excessive disruption, remains to be determined.

## FAP theranostics

### Overview

Theranostic agents are those that can be used both for imaging and therapy by substituting therapeutic isotopes for diagnostic isotopes. The theranostic potential of FAP-targeted pharmaceuticals, based on the excellent demarcation of primary and metastatic lesions in multiple cancers, has been recognized since the early phase of FAP compound development. The first-in-human theranostic application of FAPI was the administration of Y-90-FAPI-04 in a female patient with metastasized breast cancer after a preceding diagnostic FAP-PET, showing a substantial intratumoral uptake^27^. This treatment led to a significant reduction in pain medication in this patient with minimal toxicity^27^. Currently, FAPI-04 and FAPI-46, containing DOTA-chelators, which have the capability of binding Ga-68, Y-90, or Lu-177, are the compounds most widely used as theranostic FAPI ligands^34^. Major innovations include peptide compounds (Ga-68/Lu-177-FAP 2286)^38^ and dimeric FAPI derivates (Ga-68/Lu-177-DOTAGA.(SA.FAPi)_2_)^40^ as theranostic pairs. Currently published data on FAP theranostics is summarized in Table 2. Although these results are promising, they are only proof-of-concept studies performed in single institutions, and the larger validation studies are needed.

**Table 2:** Overview of selected therapeutic applications of FAP ligands

Radionuclide	Subject	Dose	Dosimetry	Results	Authors
Lu-177-FAPI-46	21 patients with advanced cancers, nonoperable or refractory to conventional therapies	1.85-4.44 GBq; median 3.7 GBq per cycle with intervals of 4 to 6 weeks between the cycles	Median absorbed dose: whole body 0.026, liver 0.136, kidney 0.886, spleen 0.02 mGy/MBq	12 cases of stable disease and 6 cases of progressive disease	Assadi et al.^241^
Y-90-FAPI-46	9 patients either with metastatic soft-tissue or bone sarcoma or with pancreatic cancer, who showed both an exhaustion of all approved therapies	3.25–5.40 GBq: median 3.8 GBq for the first cycle, and 3 patients received subsequent cycles with a median of 7.4 GBq	Median absorbed dose: kidney 0.52, bone marrow 0,04 Gy/GBq, <0.26 Gy/GBq in the lung and liver. Tumor lesions up to 2.28 (median 1.28 Gy/GBq)	4 cases of stable disease and 4 cases of progressive disease	Ferdinandus et al.^242^
Sm-153-/ Y-90-FAPI-46	1 patient with lung metastasized soft tissue sarcoma	3 cycles with cumulative 20 GBq of Sm-153- and 8 GBq of Y-90-FAPI-46	Not mentioned	Stable disease for 8 months	Kratochwil et al.^243^
Lu-177-DOTAGA.(SA.FAPi)_2_	7 patients with various cancer entities (thyroid cancer, breast cancer, paraganglioma)	0.6–1.5 GBq: median 1.48 GBq at the first cycle of treatment. Two cycles of treatment at a median interval of two months	Mean absorbed dose: whole body 0.023, liver 0.21, kidney 0.37, spleen 0.006, bone marrow 0.012 Gy/GBq	Clinical response in all patients	Ballal et al.^40^
Lu-177-FAP-2286	11 patients with metastasized pancreas, breast, rectum, and ovarian cancer	Overall 2.4–9.9 GBq, mean 5.8 GBq for each patient. 1–3 therapy cycles were performed per patient	The whole-body effective dose: 0.07 ± 0.02 (range 0.04–0.1 Gy/GBq)	Stable disease in 2 patients and progression in the other 9 patients	Baum et al.^36^

### Challenges in the development of a theranostic tracer

The main challenge in the development of a theranostic tracer is to create a compound with prolonged tumor retention, matching that of the half-life of the radioisotope^222^. This maximizes dose to the tumor while minimizing toxicity and biosafety hazards. The ideal theranostic agent is characterized by high initial tumor uptake with minimal off-target uptake and rapid clearance, thus minimizing toxicity to major organs^222^. For this reason, some compounds may be suitable for diagnostic purposes only in combination with a shorter-lived diagnostic emitter such as Ga-68^222^. For example, ^68^Ga-FAPI-02, one of the first FAPI compounds developed in 2018, showed a desirable, high internalization rate into the target cells, but due to the relatively rapid efflux and shorter intra-tumoral retention compared to other compounds e.g., FAPI-04 and FAPI-46, FAPI-02 has not been further considered for therapeutic use^27^. Given that the tumor retention time remains short, short-lived isotopes such as α-emitter Bi-213 or the β-emitter Re-188 may ultimately be more suitable to deliver higher radiation dose to the tumor than current longer-lived isotopes like Lu-177^34^. Precise dosimetric analysis with larger number of patients is also pivotal for the accurate estimation of delivered radiation dose to tumors and normal tissues.

### New trends

#### Covalent targeting

Covalent targeted radioligand (CTR) is a novel strategy for the prolonged binding of radioligands to their targets^223,224^. In this strategy, a ligand “warhead” which binds covalently to the target molecule, is conjugated to a linker and the chelated radioisotope^225^. When CTRs reach the tumor, the molecule first non-covalently binds to the target through a conventional ligand-target interaction but rapidly becomes irreversibly bound due to covalent interactions of the warhead. Other CTRs that do not bind to the target undergo rapid renal excretion, preventing off-target binding^226^, thus limiting the risk of systemic toxicity. Recently, Cui et al. developed a CTR, using FAPI-04 with an attached sulfur fluoride-based warhead^225^. This warhead reacts with the nucleophilic sites of FAP in the side chain to create covalent binding. Instead of sulfone fluoride (SF), fluorosulfate (FS) has been introduced as an alternative warhead, because it exhibits higher stability with increased cellular uptake and retention^225^. Thus, covalent binding strategies might be able to enhance the tumor uptake and retention of radioligands considerably. However, an important design criterion for CTRs is that they avoid uncontrollable ligation reactivity during circulation or in healthy tissues, which will require more development.

#### FAPI theranostics in combination with immunotherapy

It is increasingly evident that targeted radionuclide therapy might affect the immunogenicity of tumors through direct and indirect immunostimulatory effects^227–229^. In vitro studies have shown that radiation-induced DNA damage can cause the accumulation of nucleic acid fragments in the cytosol of cancer cells, leading to the stimulation of pro-inflammatory cytokines^230,231^. Increased levels of pro-inflammatory cytokines attract immune cells that may boost the immune response against the tumor^231^. Thus, radionuclide therapy has the potential to convert immunologically inactive (so-called “cold”) tumors into immunologically active (“hot”) lesions more likely to respond to immunotherapy^227,232^. Numerous preclinical studies suggest the synergistic effect of radiation and immunotherapy^233–238^. The majority of these studies assess the effect of combined radionuclide therapy with immune checkpoint inhibitors (ICIs) in melanoma, prostate, colon, or breast cancer^233–238^. So far, however, there is sparse evidence this can be extended to humans with cancer. Zboralski et al. have recently demonstrated that Lu-177-FAP-2287, a murine-targeted-FAP agent, enhanced anti-PD-1 mediated tumor growth inhibition^239^. This result offers hope that FAP-targeted radionuclide therapy in combination with ICI may be more effective than either strategy alone.

## Summary and future perspectives

The current state of the art of FAPI-PET reveals wide-ranging potential applications in oncology and non-oncologic diseases. While the exploration of many of the non-oncological indications is currently just emerging, evidence that FAPI-PET surpasses FDG-PET in many cancer types has been steadily accumulating and suggests a promising role for FAPI-PET, especially in tumors where FDG-PET has proven insensitive or non-specific. This includes hepatic, gastric, pancreatic, colorectal, lung, breast, and ovarian cancer, where the superior sensitivity of FAPI-PET compared with FDG-PET is evident. While FAPI-PET consistently demonstrates higher sensitivity than FDG in multiple malignancies, there are limitations to FAPI due its uptake in inflammation and benign fibrosis.

Currently, there are several gaps in knowledge on the performance of FAPI-PET. One such gap is how well the tracer can differentiate between benign and malignant lesions given the avidity for both. The differentiation of inflammatory from malignant lesions is one of the major issues, which needs further analysis. Some hope is offered by the observation that multi-time point imaging obtained over several hours, can demonstrate washout of activity with inflammation but plateauing or increases in cancer, however, some overlap between the two is inevitable^98,221^. Detailed histopathological and immunological evaluation of FAP expression in various inflammatory or infectious diseases might be a way to clarify this issue.

It is also now clear that FAPI uptake can vary with specific histological subtypes, particularly in diseases with low FDG avidity. Several comparative studies have shown that tumors with known low FDG avidity such as lung adenocarcinoma or signet-ring gastric carcinoma, are most benefitted by the use of FAPI-PET^91,115^. Larger clinical studies stratified by histologic subtype and FDG uptake, e.g., invasive lobular breast cancers^130,131^ or multiple sarcoma subtypes, are needed to fully characterize the role of FAPI-PET more precisely.

A final gap in knowledge is to what extent FAPI-PET uptake reflects prognostic information and whether it is a possible surrogate for disease outcomes. Several studies have addressed the prognostic impact of FAPI-PET, showing that the FAP signal intensity correlated with the clinical severity and disease extent, which in some cases is predictive of disease outcome^74,92,95,102,103,163,192^, however, more work is needed.

The future of FAPI-PET is quite promising. However, we also need to consider other radionuclidic forms of FAPI imaging including ^99m^Tc-labeled FAPI-SPECT imaging, which although lower in resolution, will be more flexible and cost-effective and perhaps more useful in environments with fewer PET scanners^31,33,240^. At the other end of the cost spectrum, FAPI-PET/MRI may also lead to increased flexibility in clinical choice, as MRI is regarded as the standard radiographic method in several cancers (e.g., liver, brain, breast cancer, or soft tissue sarcomas).

To summarize, well-conducted clinical trials with sufficiently large cohorts of patients to allow for valid subgroup analysis, detailed analysis of histological subtypes as well as the correlation with longitudinal clinical outcome, will be important in defining the future of FAPI-PET. New developments in super-sensitive whole-body PET technology will also, no doubt, advance the utility of FAPI imaging. Finally, the potential to directly target FAP expression either by FAP-targeted drugs or radioligand therapy, opens further opportunities for image-directed therapy in cancer and non-cancer applications. FAPI-PET will continue to be an exciting area of research in the coming years.
